# A gene bank's collection of genetic diversity among minor chicken breeds^1^

**DOI:** 10.1016/j.psj.2023.102827

**Published:** 2023-06-01

**Authors:** H.D. Blackburn, B.C. Krehbiel

**Affiliations:** ⁎ National Animal Germplasm Program, Agricultural Research Service, United States Department of Agriculture, Fort Collins, CO 80521, USA; † Colorado State University, Fort Collins, CO 80521, USA

**Keywords:** chicken, genetic diversity, genetic conservation, gene bank, rare breeds

## Abstract

Genetic differences among heritage or fancier breeds of chickens have not been quantified in the United States. Gene banks collecting germplasm for conserving these breeds need this information as do breeders and companies raising them. Our goal was to evaluate genetic diversity of 10 heritage/fancier chicken breeds that are a component of the national collection and to use this information to establish a baseline of their genetic diversity and future conservation efforts. Breeds could be broadly classified as European, Asian, Mediterranean, and United States (**US**) in origin. The US breeds were composite breeds developed between the 1849 and 1935. Animals (*n* = 24–31 per breed) were sampled for DNA analysis from 2 or 3 hatcheries per breed and a total of 8 hatcheries. The hatcheries were assumed to maintain and breed their own populations of the studied breeds. Effective population sizes ranged from 47 to 145 and used to estimate probabilities of extinction for a 50-generation timeline. It was determined that Crevecoeur and Aseel had a probability of extinction that exceeded 40%, the remaining 8 breeds had probabilities of <28%. ADMIXTURE analysis indicated the minimal CV corresponded to 9 populations. In that analysis New Hampshire and Rhode Island Red were classified as the same population, which was not unusual given that New Hampshire was developed as a subpopulation of Rhode Island Red. Crevecoeur and Buttercup were the 2 most genetically divergent breeds based on pairwise *F*_st_ among the breeds and principal component analysis, which was supported by the ADMIXTURE results. Inbreeding coefficients computed from genomic information was lowest for Crevecoeur, Rhode Island Red, Buttercup, and Andalusian (0.8–2.6%), while New Hampshire, Buckeye, and Aseel were highest (12.8–14.3%). Within breed *F*_st_ among hatcheries supplying animals for sampling generally indicated a genetic structure was present on a breed-by-breed basis. Genetic relationships within hatchery were also computed for each breed. Several of the hatcheries had sent samples that suggested genetic relationships as high as half-sibs while several others had genetic relationships closer to first cousins. We conclude that the chicken breeds evaluated have substantial genetic variability within the in situ populations and the gene bank has captured this diversity for future use.

## INTRODUCTION

The importance of genetic resources for industry and scientific use has been discussed as a component of USDA's 2018 to 2027 genome to phenome blueprint (Rexroad et al., 2019). Since 2000 the national gene bank for livestock has been developing germplasm and tissue collections to conserve poultry genetic resources and supports USDA's goals. For several countries, with similar goals, these collections have become large in terms of numbers of breeds, animals, and samples. As examples, in Europe 11 gene banks (excluding France) report having 68 chicken breeds cryopreserved (EUGENA https://www.eugena-erfp.net/en/searchen-gb), while France's gene bank reports 73 breeds (CRB-ANIM https://www.crb-anim.fr/crb-anim_eng/), and in the United States there are 23 breeds and 145 subpopulations (principally composed of research lines) (https://agrin.ars.usda.gov/collection_overview_page_dev?language=EN&record_source=US). In this study, we use the US collection to quantify the genetic variation that has been acquired in the collection and genetic differences among noncommercial chicken breeds which have not been addressed previously.

Prior to this study, genetic diversity assessments of US heritage/fancier (**HF**) chicken breeds have not been widely performed. The breeds studied were either imported or developed as new composite breeds after entering the United States. Those breeds representing the original imported breed were derived from countries in Europe or Asia. Domestication, historic routes of dispersal, and early breed formation have been discussed (Tixier-Boichard et al., 2011; Lawal et al., 2020; Lawal and Hanotte, 2021). The principal route of migration to the western hemisphere appears to have been via Atlantic trade routes where Asian and European breeds were exchanged (Herrera et al., 2020). The popularity of the US composite breeds is variable. Breeds like Rhode Island Red or Plymouth Rock have become globally important (FAO, 2023) due to their performance levels for economically important traits, but others have not gained in popularity (e.g., Buckeye). The number of progenitor breeds and their composition in the composite breeds are not well documented. But for example, Plymouth Rock is thought to have as many as 6 progenitor breeds (Guo et al., 2019). Furthermore, development of composite populations and within breed selection has been a common practice in the development of various commercial broilers and layers as well (Delany, 2003). However, such populations may have had a contraction of genetic diversity (Muir et al., 2008). But, more recent findings by Malomane et al. (2019) suggested that diversity of commercial broiler populations is similar to wild populations. But the status of HF breeds, as in this study, is largely unknown.

The current US chicken industry can be divided into 3 segments: commercial broilers, commercial layers (both with their well-structured breeding programs), and the smaller HF breed sector that does not employ high levels of selection nor industrial concentration. Reduced numbers of breeders for HF breeds have created a marketing niche for commercial hatcheries that maintain multiple lines or breeds and then sell eggs or day-old chicks regionally and/or nationally. The populations maintained by such hatcheries may represent lines bred by the hatchery over time and/or the sourcing of breeding stock from other breeders and then multiplying those genetics. It is not known if multiple hatcheries source breeding stock from the same source.

Given the importance of this hatchery system to the maintenance and distribution of HF breeds it is of interest to quantify the genetic diversity they maintain and offer for sale. Therefore, in the present study we report an evaluation of 10 HF chicken breeds all from nonindustrial populations but sourced from 8 commercial hatcheries and 1 university. The goal is to better understand the genetic diversity of the breeds evaluated, differences among hatchery populations, and assess the use of the information in conservation activities.

## MATERIALS AND METHODS

### Breeds Studied

Breeds, country of origin, number of hatcheries sampled, number of animals sampled, and conservation classification (FAO, 2023 https://www.fao.org/dad-is/data/en/) are provided in Table 1. All breeds developed in the United States are composite breeds derived from 3 to 6 different breeds that were imported to the United States from different geographic locations (Supplementary Table S1), for example, Rhode Island Red (**RIR**) is thought to have been formed by using Malay (South Asia), Brown Leghorn (Tuscany in Italy), and potentially Java (Java) and Shanghai (China). While Buckeye is a 3-way cross that used Black Breasted Red Game X (Buff Cochin X Barred Plymouth Rock). Guo et al. (2019) is one of the few studies documenting the contribution of various breeds to the formation of the Plymouth Rock. The Andalusian, Aseel, Buckeye, Buttercup, Crevecoeur, and Phoenix are considered to be at varying levels of genetic risk (Table 1, FAO, 2023). Plymouth Rock and Rhode Island Red are important breeds in the layer or broiler industries, but our samples were not sourced and considered a different subpopulation with only ancestral genetic linkages to highly selected industrial populations. There is no reported gene flow between the industrial and fancier/heritage populations of these 2 breeds.

**Table 1:** Country of origin, year imported to the United States, general category, conservation status, hatcheries sampled, and number of animals genotyped and used in the study for each breed.

Breed	Country of breed origin	Year(s) imported or developed	Breed category	FAO conservation category/US status^1^	Hatcheries sourced	Number sampled
Andalusian	Spain	1850–1855	Mediterranean	At risk—globally/vulnerable	3	29
Aseel	India	1887	AOSB-Asian^2^	At risk—globally/endangered maintained	3	27
Buckeye	USA	1896	American	At risk—globally/vulnerable	3	30
Buttercup	Italy	1835	Mediterranean	At risk—globally/vulnerable	2	24
Crevecoeur	France	1852–1870	Continental	At risk—globally/endangered maintained	2	28
Jersey Giant	USA	1870–1890	American	Not at risk—globally/not at risk	2	24
New Hampshire	USA	1935	American	Not at risk—globally/not at risk	3	30
Phoenix	Germany/Japan	1924	AOSB-Asian^2^	At risk—globally/vulnerable	3	28
Plymouth Rock	USA	1849	American	Not at risk—globally/not at risk	2	31
Rhode Island Red	USA	1880–1890	American	Not at risk—globally/not at risk	3	26

1 Risk categories define by FAO (FAO, 2007).

2 All other standard breeds—Asian origin (as designated by American Poultry Association).

### Sample Acquisition

DNA for this study was extracted from cryopreserved tissues that were collected by the National Animal Germplasm Program (**NAGP**) as part of its breed conservation efforts. No animals were sampled specifically for this study. Eight different hatcheries and 1 university were used as sources of samples for this evaluation. Each breed is represented by sourcing samples from 2 to 3 hatcheries. Each hatchery provided samples from 1 to 5 breeds. Typically, no one hatchery maintains and sells all chicken breeds. Hatchery geographic distribution extended from the Midwest to California and from Wisconsin to the southern USA. The Wisconsin source was the University of Wisconsin that only supplied New Hampshire samples. This source and population were selected because they were highly inbred and therefore could serve as an additional benchmark to compare other lines and breeds.

### Genotyping

All animals sampled were genotyped with the Affymetrix Axiom Chicken Beadchip (580,961 SNP; Kranis et al., 2013). Quality control filtering was applied to the 580,961 SNP genotypes: minor allele frequency (**MAF**) <0.05, sample call rate <0.95, and Hardy-Weinberg equilibrium (**HWE**) <0.001. Additionally, the sex chromosomes and ambiguous marker information were removed from the dataset. After filtering 76,760 SNP were used in the analysis. Admixture 1.3 (Alexander et al., 2009) was performed on the populations to determine population substructure. Alexander et al. (2009) assessed the maximum likelihood approach developed for the software provided a useful approximation of linkage disequilibrium (**LD**) therefore in running ADMIXTURE no additional LD filtering was performed. In preliminary ADMIXTURE runs, it became apparent that 14 chickens (*n* = 8 Plymouth Rock; *n* = 6 Aseel) were crossbred and were removed from the dataset. This preliminary analysis also showed 2 Buckeyes where Phoenix or Rhode Island Red they were therefore reassigned to Phoenix and Rhode Island Red. The software used cross-validation (**CV**) error for values of *K* from 1 through 10 with 10 repetitions for each value of *K* being run. The lowest CV error represented the appropriate number of subpopulations for evaluation. StructurePlot (Ramasamy et al., 2014) was used to illustrate the proportional assignments of chickens to the varying clusters.

Among and within breeds and hatcheries, principal component analysis (**PCA**) genomic relationship matrix, *F*_st_, and baseline genetic parameters of observed and expected homozygosity, minor allele frequency, were performed using SNP & Variation Suite (Golden Helix Inc., Bozeman, Montana, USA). Additional pruning of the data was performed to adjust for linkage disequilibrium using a window size, step size, and threshold of 50, 5, 0.2, respectively, which left 75,558 SNP in the analyses. Additionally, inbreeding coefficients were calculated for the breeds and hatcheries within breed.

Effective population size and its trend over generations were computed with SNeP software as described by Barbato et al. (2015). With *N*_e_ computed, a probability of extinction (*P*_ext_) after 50 generations could be calculated as described by Ollivier et al. (2005), and has the form:

$$P_{\text{ext}}=1-\text{exp}\left(-50/2N_{\text{e}}\right).$$

This equation provides insight into the potential extinction based only upon genetic factors (e.g., mutation rates) and not social or market perspectives. Therefore, it provides information that is useful in current *in situ* management of breeds. Of course, with samples secured in the gene bank extinction of a breed has been rendered something of an obsolete concept.

Within breed analysis of inbreeding and genetic relationships (by hatchery) were computed using the genomic relationship matrix (**GRM**) available in the Golden Helix SVS software package.

## RESULTS

Expected and observed homozygosity ranged from a low of 0.08 for Crevecoeur to 0.17 for New Hampshire. The lowest levels of Ho and He and conversely the highest fixed alleles were for Buttercup and Crevecoeur (Supplementary Figure 1).

### Principal Components

**Figure 1:**
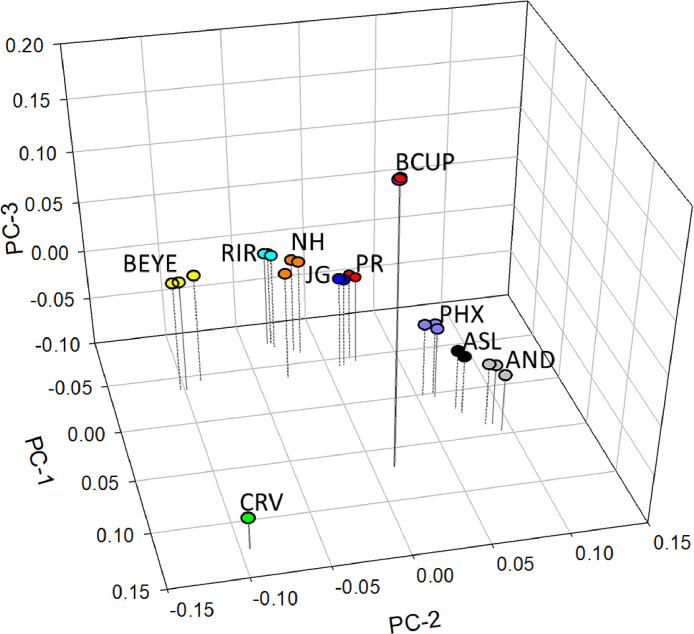
Principal component analysis of the breeds evaluated, where the variation explained by principal components PC-1, PC-2, and PC-3 was 8.26, 5.62, and 5.5%, respectively. Where BEYE = Buckeye; RIR = Rhode Island Red; NH = New Hampshire; JG = Jersey Giant; PR = Plymouth Rock; PHX = Phoenix; ASL = Aseel; AND = Andalusian; BCUP = Buttercup; and CRV = Crevecoeur. Each circle for a breed represents a different hatchery (Buttercup and Crevecoeur hatcheries overlap).

Figure 1 provides a relative view of how the breeds and hatcheries within breed are associated with one another. The first principal component suggested 3 major groupings of breeds corresponding to breeds originating in the United States, Asia, and Europe. Crevecoeur and Buttercup placement were the most distant from other breeds tested. The Spanish Andalusian was closely placed next to the Asian Aseel and Phoenix. The second principal component separated the US breeds from each other as well as the Asian grouping. Among the US breeds Buckeye were the most distinct in their placement. Principal component 3 placed Buttercup and Crevecoeur at opposite ends of the range with United States and Asian populations being intermediate.

Hatchery differences within breed are evident by the placement of a breed's subpopulations (hatcheries) on Figure 1. Hatcheries supplying Crevecoeur and Buttercup lacked separation suggesting little difference in these 2 breed's subpopulations.

### Admixture Analysis

**Figure 2:**
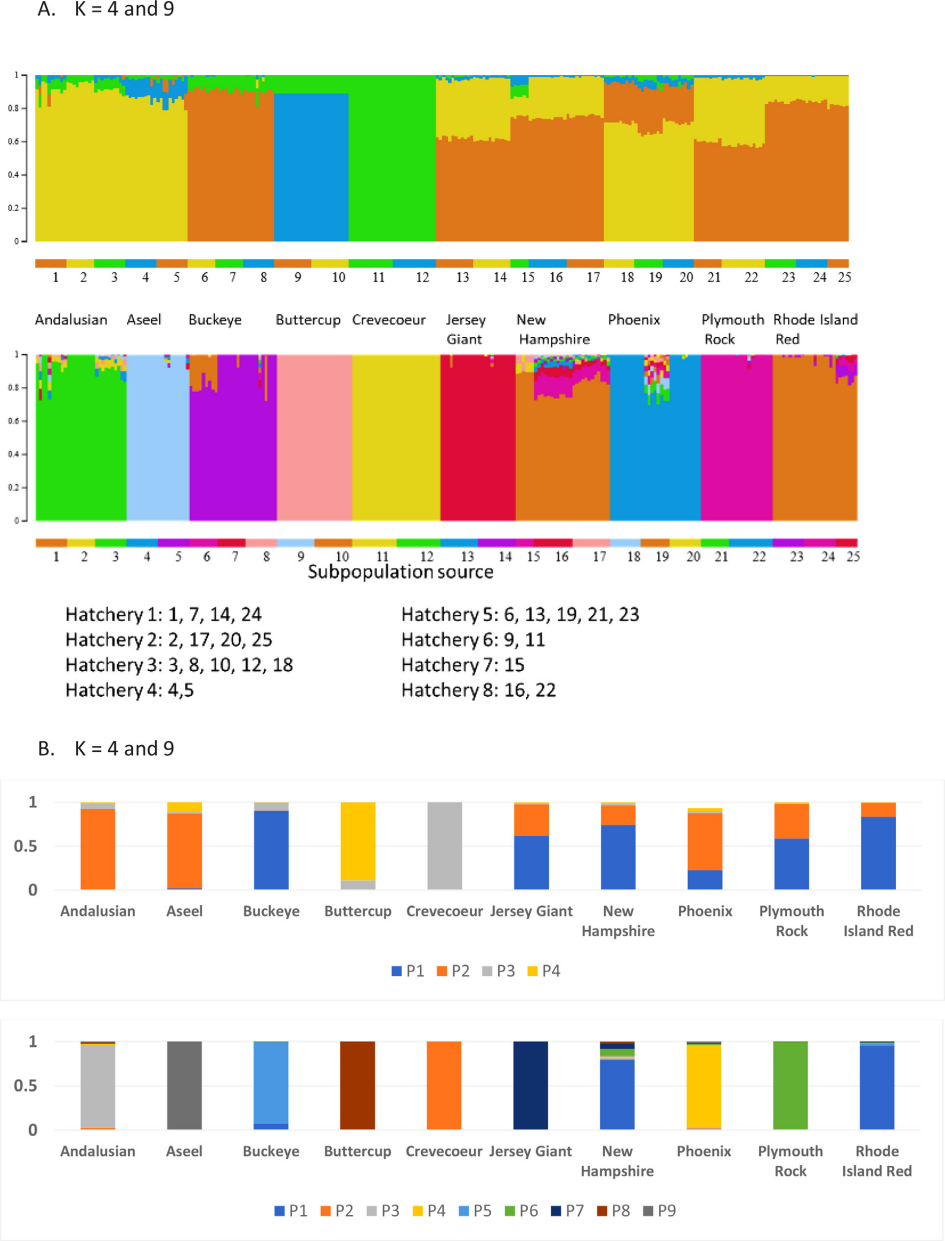
ADMIXTURE analysis using K of 4 and 9. (A) Provides individual animal cluster assignments, breed and hatchery where samples were sourced. (B) Shows cluster assignments as breed averages.

Population structure was explored with ADMIXTURE with clusters ranging from 2 to 10. CV error was used to identify values for *K* for evaluation (Supplementary Figure 2). We present *K* = 4 and *K* = 9 (Figure 2) to show the development of the clusters and breed assignments. Specifically, *K* = 4 provides insight into the ancestral formation of the breeds evaluated. All breeds except Crevecoeur showed some level of admixture. The Aseel and Andalusian shared a high proportion of their assignment in the same cluster despite the distance of their geographic origin. Breeds developed in the United States were admixed with clusters where Aseel and Andalusian and Buckeye had an assignment of >80%. Interestingly the split of proportional assignments were similar for Jersey Giant, New Hampshire, Plymouth Rock, and Rhode Island Red.

When *K* = 9 each breed had a unique cluster assignment, except Rhode Island Red and New Hampshire (Figure 2) which shared the same cluster. Given New Hampshire were developed as a subpopulation of Rhode Island Red the association between the 2 breeds seems appropriate. At *K* = 9 hatchery effects can be observed. Three hatcheries were found to have produced 6 admixed populations that were greater than 10%. Subpopulations 3, 6, 16, 17, 19, 25 bred at 4 hatcheries (Figure 2) had the highest levels of admixture. The New Hampshire in subpopulation 16 and 17 had higher admixed levels with the Plymouth Rock. Subpopulation 6, Buckeye, was predominantly admixed with Rhode Island Red or New Hampshire. Phoenix subpopulation 19 appeared to be admixed with several breeds in the analysis.

### *F*_st_ Among Populations

Structure among breeds was tested by computing pairwise *F*_st_ (Table 2). Most pairwise comparisons showed substantial differences among tested breeds. The greatest differences in *F*_st_ occurred when comparing Buttercup and Crevecoeur to the other breeds evaluated and the highest *F*_st_ occurred in comparing these 2 breeds with each other (0.30). The closest pairings (*F*_st_ < 0.10) suggesting a weaker genetic structure between New Hampshire and Rhode Island Red and Plymouth Rock.

**Table 2:** Pairwise *F*_st_ among breeds, lowest values in bold and highest are in italic.

Breed	Andalusian Blue	Aseel	Buckeye	Buttercup	Crevecoeur	Jersey Giant	New Hampshire	Phoenix	Plymouth Rock White	Rhode Island Red
Andalusian Blue	–									
Aseel	0.18	–								
Buckeye	0.17	0.20	–							
Buttercup	0.21	*0.25*	0.23	–						
Crevecoeur	0.22	*0.27*	*0.25*	*0.30*	–					
Jersey Giant	0.16	0.18	0.15	0.22	*0.25*	–				
New Hampshire	0.13	0.15	0.11	0.18	0.21	0.11	–			
Phoenix	0.14	0.16	0.15	0.20	0.22	0.14	0.11	–		
Plymouth Rock White	0.14	0.16	0.14	0.20	0.23	0.12	**0.09**	0.12	–	
Rhode Island Red	0.14	0.16	0.11	0.20	0.23	0.12	**0.06**	0.12	0.10	–

### Effective Population Size

**Figure 3:**
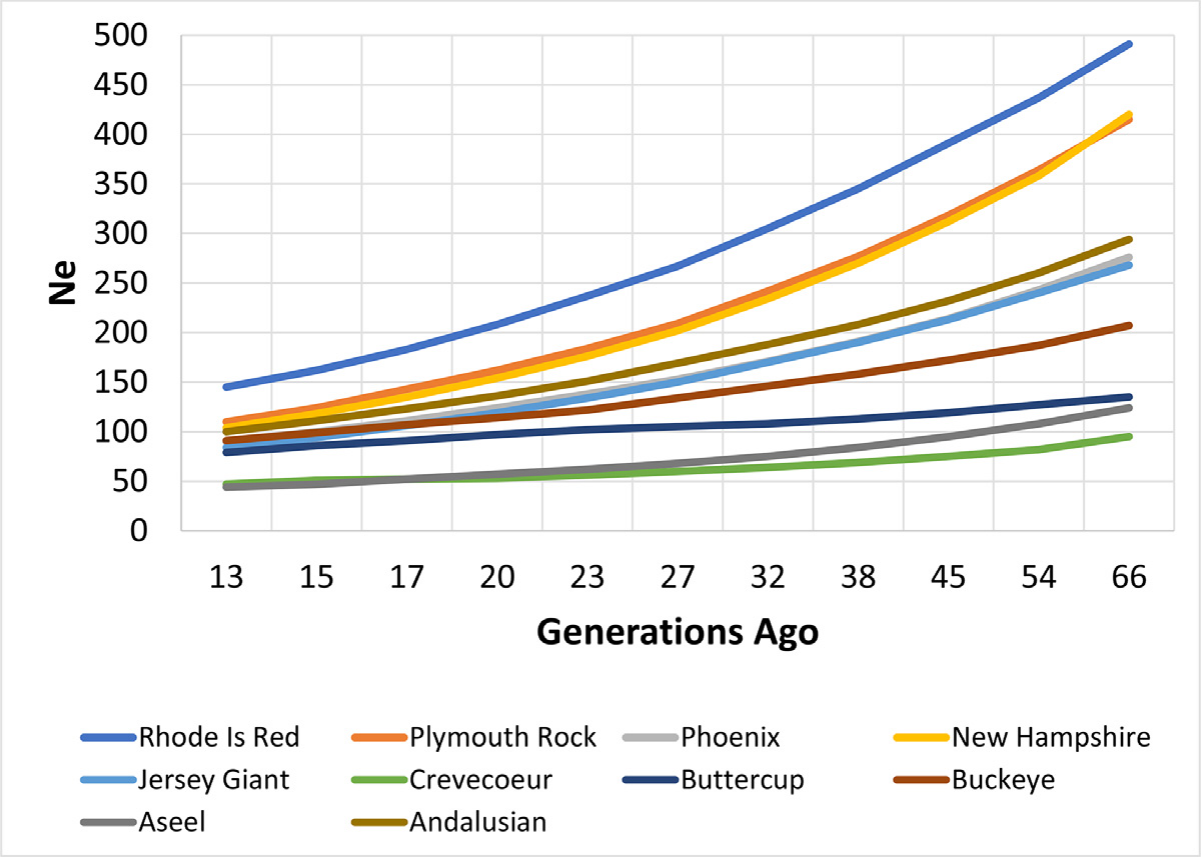
Change in effective population size (*N*_e_) over generations for 10 chicken breeds.

Effective population size (*N*_e_) was computed for each breed (Figure 3). Aseel and Crevecoeur had the smallest *N*_e_ at 44 and 47 animals, respectively, while Rhode Island Red had the largest (*N*_e_ = 145). The remaining breeds’ *N*_e_ ranged from 79 to 110 animals. While Aseel, Crevecoeur, and Buttercup had the lowest *N*_e_ their curves had the slowest rate of decay (Figure 3). Once *N*_e_ was computed per breed it was used to estimate the probability of extinction in 50 generations (Ollivier et al., 2005). Accordingly, Crevecoeur and Aseel had the highest probabilities of extinction (>40%), while the remaining breeds all had probabilities of <28%.

### Within Breed Analysis

Genetic diversity within breeds was evaluated using *F*_st_ among hatcheries, computing inbreeding for each breed, and quantifying the genetic relationships among hatcheries within breed. *F*_st_ ranged from 0.0 to 0.26 (Table 3). No population structure existed among the hatcheries supplying samples from Buttercup and Crevecoeur (*F*_st_ = 0.0). The remaining breeds showed varying levels of subpopulation differentiation (0.01–0.27). Highest *F*_st_ (0.26 and 0.27) were associated with the university hatchery that supplied New Hampshire.

**Table 3:** Genetic differences among hatchery populations within breed as measured by *F*_st_.

Breed	Hatchery							
Hatchery	1	2	3	4	5	6	7	8
Aseel								
4								
Andalusian								
1	—	0.06	0.09	—	—	—	—	—
2			0.15					
3								
Buckeye								
1	—	—	0.07	—	0.10	—	—	—
3					0.08			
5								
Buttercup								
3	—	—	—	—	—	0.00	—	—
6								
Crevecoeur								
3	—	—	—	—	—	0.00	—	—
6								
Jersey Giant								
1	—	—	—	—	0.15	—	—	—
5								
New Hampshire								
2	—	—	—	—	—	—	0.27	0.09
7								0.26
8								
Phoenix								
2	—	—	0.18	—	0.12	—	—	—
3					0.12			
5								
Plymouth Rock								
5	—	—	—	—	—	—	—	
8								0.09
Rhode Island Red								
1	—	0.07	—	—	0.01	—	—	—
2					0.06			
5								

Inbreeding levels for all study animals were computed with the genomic relationship matrix (**GRM**; Golden Helix,) then averaged by inbred animals measured within breed, and all animals within a breed. Andalusian, Buttercup, Crevecoeur, and Rhode Island Red had inbreeding levels of less than 3% (Table 4). Aseel (13.6%) and Buckeye (14.3%) had the highest average inbreeding levels. Average inbreeding for New Hampshire was also high (12.8%) but the high genetic relationship among the university hatchery subpopulation increased the average for the entire breed estimate. For the remaining 7 breeds average inbreeding levels are low and typical of levels observed in other species. For example, Srikanth et al. (2022) reported an average inbreeding level of 11% for US Jersey dairy cattle using the GRM.

**Table 4:** Percent of inbred animals, average inbreeding of inbred animals, and average inbreeding per breed.

Breed	Inbred animals (% of total inbred)	Average inbreeding of inbred animals, %	Average inbreeding for breed
Aseel	10 (37.0)	36.7	13.6
Jersey Giant	22 (91.7)	7.3	6.7
Andalusian	4 (13.8)	18.9	2.6
Buckeye	15 (50.0)	28.5	14.3
Buttercup	4 (16.7)	4.8	0.8
Crevecoeur	8 (28.6)	6.0	1.7
New Hampshire	29 (96.7)	13.3	12.8
Phoenix	26 (92.8)	9.8	9.1
Rhode Island Red	7 (26.9)	7.2	1.9
Plymouth Rock	21 (67.7)	7.9	5.4

Average genetic relationships were calculated within each hatchery and for the breed. Average genetic relationships for breeds ranged from 0.058 to 0.240 (Table 5) with Rhode Island Red (0.058) having the lowest relationship (0.058) and Phoenix (0.243) and New Hampshire (0.205) the highest. The New Hampshire genetic relationship was influenced by hatchery 7 which was the university population. Across breeds, while some hatcheries had high genetic relationships within a breed it was not consistent when compared to other hatcheries providing the same breed.

**Table 5:** Genomic relationships for breeds and hatcheries within breed.

Breed/breed average	Hatchery #	Genetic relationship
Aseel	5	0.643
	4	0.089
Breed average		0.167
Black Jersey Giant	1	0.165
	5	0.172
Breed average		0.168
Blue Andalusian	1	0.119
	2	0.170
	3	0.220
Breed average		0.163
Buckeye	1	0.130
	5	0.109
	3	0.079
Breed average		0.096
Buttercup	3	0.053
	7	0.063
Breed average		0.0472
Crevecoeur	3	0.099
	5	0.114
Breed average		0.109
New Hampshire	7	0.869
	8	0.120
	2	0.138
Breed average		0.205
Phoenix	3	0.237
	5	0.209
	2	0.271
Breed average		0.243
Rhode Island Red	1	0.023
	5	0.017
	2	0.131
Breed average		0.058
Plymouth Rock	5	0.261
	8	0.074
Breed average		0.151

## DISCUSSION

### Breed Analyses

Early ADMIXTURE analysis K4 (Figure 2) indicated genetic differences among United States developed and imported breeds. Imported Crevecoeur and Buttercup were distinct from other breeds, suggesting they have been maintained with little or no admixture with other breeds. Andalusian (Spain) and Aseel (India) were grouped at K4 suggesting some genetic similarity. No obvious explanation for this similarity exists given historical accounts, for while the Aseel was developed in India, the Andalusian history of development in Spain is not clear. The Phoenix was admixed with proportions divided between Aseel and Andalusian and the American composites. American breeds shared large proportions of their genetic composition between the Aseel and Andalusian group and a component that is suggestive of the emergence of an American cluster as seen in other species (Paim et al., 2020; Hay et al., 2022) or similarities among various progenitor breeds not included in the study. Buckeyes were the exception of the American group as they had no association with Aseel nor Andalusian. Rather their second largest proportion (approximately 10%) was with the Crevecoeur cluster. The large assignment of Buckeye to cluster 1 suggests a high degree of similarity to both Plymouth Rock and Rhode Island Red; both are believed to have contributed at varying levels to the Buckeye's formation (Ekarius, 2007).

All breeds were distinct from each other when *K* = 9, except for New Hampshire and Rhode Island Red which were clustered together and matched oral histories of New Hampshire being developed from Rhode Island Red (Ekarius, 2007). The rest of the American composites have become unique and separate breeds in comparison to K4. The Buckeye does exhibit about 8% admixture with the Rhode Island Red cluster.

Also, at *K* = 9 several hatcheries had higher levels of admixture for various breeds (Figure 2). Specifically, Hatcheries: 2 (New Hampshire and Rhode Island Red); 5 (Aseel and Buckeye), 3 (Andalusian), and 8 (New Hampshire). Interestingly all New Hampshire samples showed some of the highest proportions of admixture, but not all to the same populations. For example, New Hampshire from hatchery 7 (the university) had proportional assignments to Buttercup and Crevecoeur, this hatchery also had the highest genetic relationship among breeds sampled. The other 2 hatcheries showed relatively high levels of admixture with Plymouth Rock which could be a result of crossbreeding by the hatcheries. Buckeye from hatchery 5 also exhibited admixture with Rhode Island Red and New Hampshire.

In large part principal component analysis supported *K* = 4 and *K* = 9 results of ADMIXTURE. New Hampshire was in proximity to Rhode Island Red (Heidaritabar et al., 2022) and Jersey Giant which was closely placed to Plymouth Rock. The positioning of Jersey Giant and Plymouth Rock may be due to the similarity of progenitor breeds (Table 1). Principal component 1 (8.26%) broadly separated the breeds by United States, Asian, and European breeds. The Crevecoeur and Rhode Island Red were at the extremes of PC1. Principal component 2, explaining 5.61% of the variation, further differentiated the breeds with Crevecoeur and Andalusian at the extreme ranges. Interestingly, among the US developed breeds PC2 suggested that Buckeye has drifted from the other composite breeds, this may have been due to a relatively small founder population which increased the rate of genetic drift as noted in other species (Blackburn et al., 2014). The Buckeye becoming more distinct from other American composites confirms the *K* = 9 results of ADMIXTURE. Also among the US composites the Buckeye had the smallest effective population size which suggests genetic drift may have had an effect (Falconer and Mackay, 1996). Principal component 3 (5.5%) further differentiated the Crevecoeur and Buttercup.

Fixation index (*F*_st_) was used to differentiate population structure of the breeds evaluated and ranged from 0.06 to 0.30, similar ranges have been reported for British, Rwandan, and French chicken breeds (Wilkinson et al., 2012; Habimana et al., 2020; Restoux et al., 2022). Structure did exist among the breeds at varying levels. In agreement with PCA and ADMIXTURE the Crevecoeur and Buttercup were the most different from the breeds evaluated. Breeds showing the least structure were the New Hampshire, Plymouth Rock, and Rhode Island Red which ranged from 0.06 to 0.10. The relative closeness of Plymouth Rock and Rhode Island Red may be due to similar progenitor breeds (Table S1). Similar ranges in *F*_st_ have been reported for breeds considered to be quite distinct (Faria et al., 2019 ; Zhang et al., 2018). However, Brekke et al. (2020) reported an *F*_st_ of 0.426 for Plymouth Rock and Rhode Island Red but they also reported these breeds to have inbreeding levels that exceeded 50% (vs. <6% in this study) which could explain the reported differences. The levels of *F*_st_ for US developed composite breeds, Rhode Island Red, Plymouth Rock, New Hampshire, Jersey Giant, and Buckeye, suggest a level of distinctness and structure has been obtained since breed formation. Paim et al. (2020) demonstrated this with Brangus cattle and we assume similar processes occurred in the formation of these chicken breeds especially since many more generations have occurred since the chicken breeds’ formation vs. Brangus.

Generally, within breed effective population size suggests sufficient genetic diversity exists among these breeds. Only Aseel and Crevecoeur fell below the FAO (2012) recommendation that *N*_e_ be >50; however they did have an *N*_e_ of 47. Additionally, Buckeye and Buttercup have *N*_e_ > 50 even though they have an “at risk” classification. This finding suggests that while they are classified nationally and internationally as being “at risk” (based on census data) their genetic diversity is larger than might be expected. The large *N*_e_ for Rhode Island Red, New Hampshire, and Plymouth Rock suggest substantial levels of genetic diversity exists among these breeds thereby warranting the “not at risk” rating (Table 1). The remaining 5 breeds while being classified as “at risk” have *N*_e_ ranging from 86 to 111 also drawing into question their “at risk” rating. In addition, this suggests a relatively large amount of genetic variability is available for breeders to utilize, which was also observed in British and French breeds (Wilkinson et al., 2012; Restoux et al., 2022). Among breeds the decay curves for *N*_e_ differed (Figure 3). Aseel, Crevecoeur, and Buttercup had flatter curves suggesting the loss of *N*_e_ per generation was slower than breeds with larger *N*_e_, for example, Rhode Island Red. A possible explanation may be a sex ratio nearing 1:1 which can serve to maintain *N*_e_ (Falconer and Mackay, 1996). Using *N*_e_ to compute probability of extinction, from the perspective of in situ population management there would appear to be a need for Aseel and Crevecoeur populations (both with *P*_ext_ > 0.40) to be more intensively managed from a genetic conservation perspective. Despite *N*_e_ > 50 for Jersey Giant, Phoenix, Buckeye, and Andalusian their probabilities of extinction after 50 generations ranged from 0.22 to 0.27 suggesting they may need increased efforts to maintain genetic diversity. However, given the general structure of heritage/fancier breeding programs concerted efforts may not be possible. Given this situation having adequate tissues collected in the gene bank will help maintain a broader range of diversity for use if the population continues to contract or the need arises to reconstitute any of these breeds.

### Within Breed Analysis

Fixation index (*F*_st_) was used to compare subpopulations within sampled breeds. Generally, some substructure existed within most breeds as demonstrated by Wilkinson et al. (2012), but there was a lack of substructure among the hatcheries for Crevecoeur, Aseel, Buttercup, and 2 of the 3 hatcheries where Rhode Island Red were sourced (Table 3).

Among the other 6 breeds the populations appear distinctive with *F*_st_ greater than what was found by Zhang et al. (2018) in several Chinese breeds. Genetic relationships were also computed (Table 5) for each breed and within hatchery. As anticipated the New Hampshire line maintained by a university (hatchery 7) showed the highest within line relationship (0.869) vs. the other hatcheries (0.120 and 0.138). For several breeds individual hatcheries had within hatchery genetic relationships of at or near being half-sibs, this was particularly the case for all the Phoenix hatcheries, plus one hatchery supplying Andalusian and Plymouth Rock. For several breeds hatcheries supplied animals that had genetic relationships suggestive of being first cousins suggesting the use of half-sibs in creating chicks that were sold.

### Inbreeding Levels

Inbreeding levels varied among breeds and ranged from 0.8 to 14.3% using genomic data (Table 4). The percent of inbred animals within a breed ranged from 13.4 to 96.7%. Andalusian and Buttercup had the lowest percent of inbred animals while Jersey Giant, New Hampshire, and Phoenix were the highest. While the breed average for inbreeding is not inordinately high the presence of animals within a breed with high levels of inbreeding suggest a need to plan matings to avoid unnecessary increases, which suggests greater emphasis on maintaining pedigrees and computing genetic relationships. We note that the relatively low levels of inbreeding for Buttercup and Crevecoeur are juxtaposed to the high level of fixed alleles for these breeds. While the results are possible it suggests the need for further exploration.

One objective was to evaluate the genetic diversity among various hatcheries that are a potential source of breeding chickens to heritage breed producers. This was accomplished by using the genomic relationship matrix within breed. The highest within population genetic relationship was for New Hampshire obtained from a university (hatchery 7) research population and Aseel from hatchery 5 (Table 5). The hatcheries sampled for Rhode Island Red and Buttercup all had relatively low within hatchery genetic relationships. Interestingly, all hatcheries providing samples from Phoenix and one hatchery for Plymouth Rock had genetic relationships at the half-sib level, suggesting small breeding populations or a biased sampling of chicks.

### General Discussion

This study suggests most breeds evaluated do not exhibit a loss of genetic diversity. The differences among the subpopulations within a breed support this conclusion based on the principal component analysis and within breed *F*_st_ analysis. While some inbreeding levels are relatively high, they most likely are not at a level that will depress performance on a breed wide scale. Furthermore, inbreeding levels are such that they can be reversed or slowed as demonstrated with Hereford cattle (Cleveland et al., 2005). However, breeders raising Aseel, Buckeye, and New Hampshire should consider implementing breeding programs that minimize the rate of inbreeding.

Buttercup and Phoenix are reported to be “at risk” in Italy and Germany, respectively, and globally (FAO, https://www.fao.org/dad-is/data/en/), while the status of Crevecoeur in France and globally is unknown (FAO, DAD-IS). We found Buttercup, Phoenix, and Crevecoeur in the United States genetically distinct from the other breeds evaluated. These breeds were imported at or over a century ago, as a result they may provide untapped potential for reintroducing genetic variability into their respective countries of origin, as suggested with goats (Paim et al., 2019). That said, Crevecoeur and Buttercup had the highest levels of fixation suggesting breeders need consolidated action to minimize further contractions of genetic diversity for those breeds.

A basic premise of our study was that relatively small commercial hatcheries would be maintaining or sourcing breeding chickens from different elite breeders, and these subpopulations facilitate maintenance of within breed genetic diversity. While the results generally support this assumption it must be recognized that the hatcheries, and breeds sampled from them, are not a full sampling of the breeds studied. We further assume there are more purebred flocks within the breeds studied and therefore genetic diversity could be greater than demonstrated in the present study. That said, this report provides a baseline for future monitoring of the breeds evaluated.

The samples used in this study were all sourced from the national gene bank (Blackburn, 2018; Blackburn et al., 2019) which currently holds 17,352 tissue samples from 2,192 chickens, representing 145 chicken subpopulations and 23 chicken breeds. The data suggest that the gene bank has acquired substantial genetic variation from the breeds tested. Samples from these breeds consist of ovaries, testes, semen, and other tissues that can be used to reconstitute breeds if needed using the approaches developed by Bacon et al. (1986) and Song and Silversides (2007, 2008) or extract primordial germ cells as demonstrated by Ballantyne et al. (2021). Using these types of approaches may be the most cost-effective way to conserve the genetic diversity of these breeds. Silverside et al. (2012) demonstrated using cryopreserved tissues to recover lost genetic diversity is 3 to 4 times cheaper than maintaining live populations. Therefore, given the diversity captured by the gene bank for the breeds in this study, breeders of these populations have additional flexibility to select and manage their flocks as they deem appropriate by using the collection.

## Appendix Supplementary materials

Image, application 1

## Footnotes

Supplementary material associated with this article can be found in the online version at doi:10.1016/j.psj.2023.102827.
