# Supplementary material for: A gene bank's collection of genetic diversity among minor chicken breeds
Source: Poult Sci. 2023 Jun 1;102(8):102827. doi: 10.1016/j.psj.2023.102827 (PMC10404745; doi:10.1016/j.psj.2023.102827)

Supplementary Material

Table S1. Progenitor breeds and their geographic origin for breeds developed in the United States.

Breed	Progenitor Breeds & geographic origin	Source
Plymouth Rock	Black Java – Java Island<br>Dominique - England<br>Cochin – China<br>Minor influences<br>Langshan - China<br>Brahman - China<br>Black Minorca - Spain	Guo et al., 2019<br>Ekarius, 2007
Jersey Giant	Black Java – Java Island<br>Black Langshan – China<br>Dark Brahman – China<br>Cornish - England	Ekarius, 2007
Rhode Island Red	Malay – South Asia<br>Brown Leghorn (Tuscany-Italy)<br>Java – Java Island<br>Cochin - China	Ekarius, 2007
Buckeye	Barred Plymouth Rock – United States<br>Buff Cochin – China<br>Black Breasted Red Game – England<br>Rhode Island Red – United States	Ekarius, 2007
New Hampshire	Rhode Island Red – United States	Ekarius, 2007

Supplementary Figure 1. Levels of expected homozygosity (He), observed homozygosity (Ho), percent of fixed alleles, and minor allele frequencies with frequencies ranging from 0.01 to 0.05.

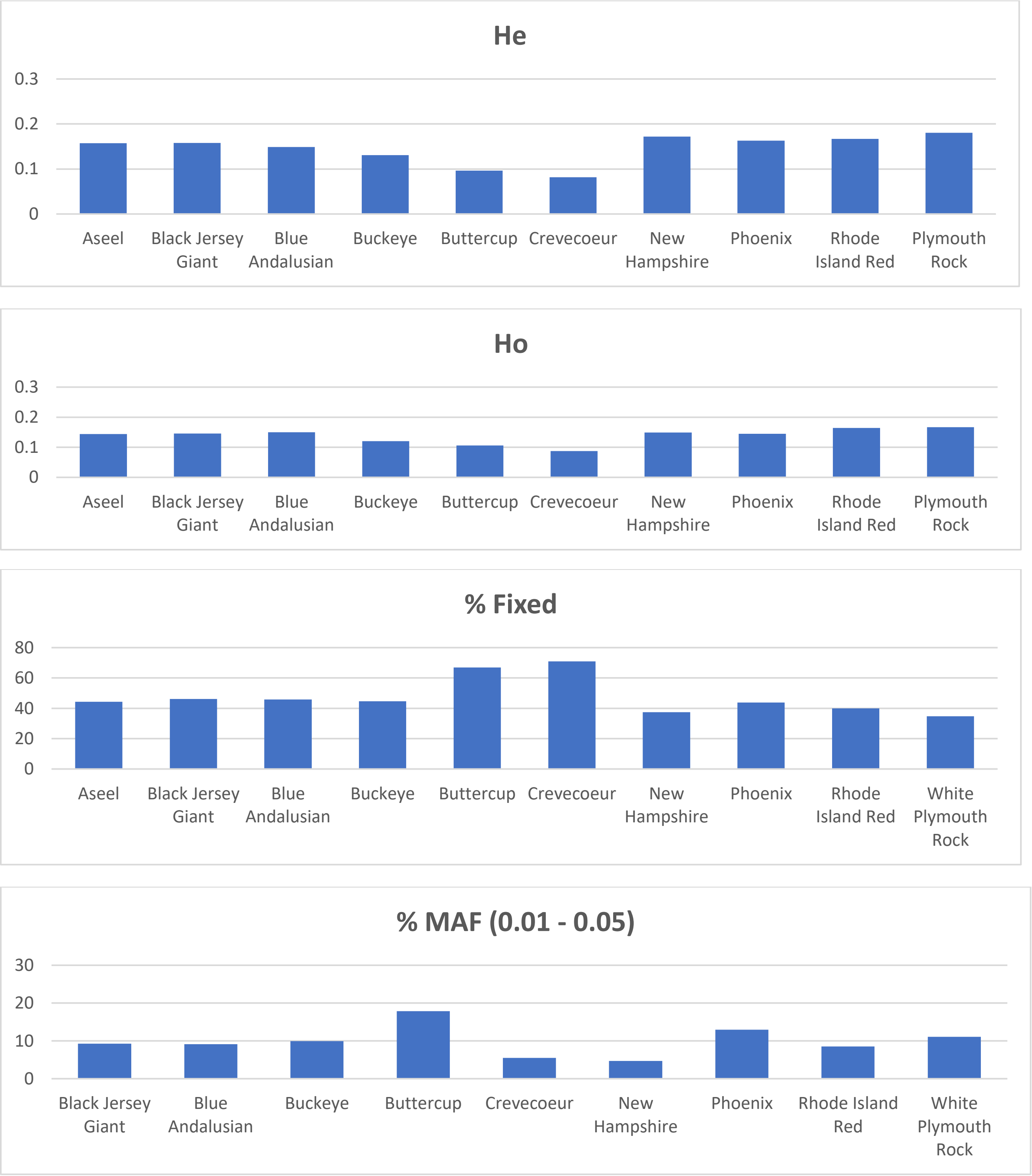

Supplementary Figure 2. The CV error for 10 repetitions of each cluster (k) used in the ADMIXTURE analysis.

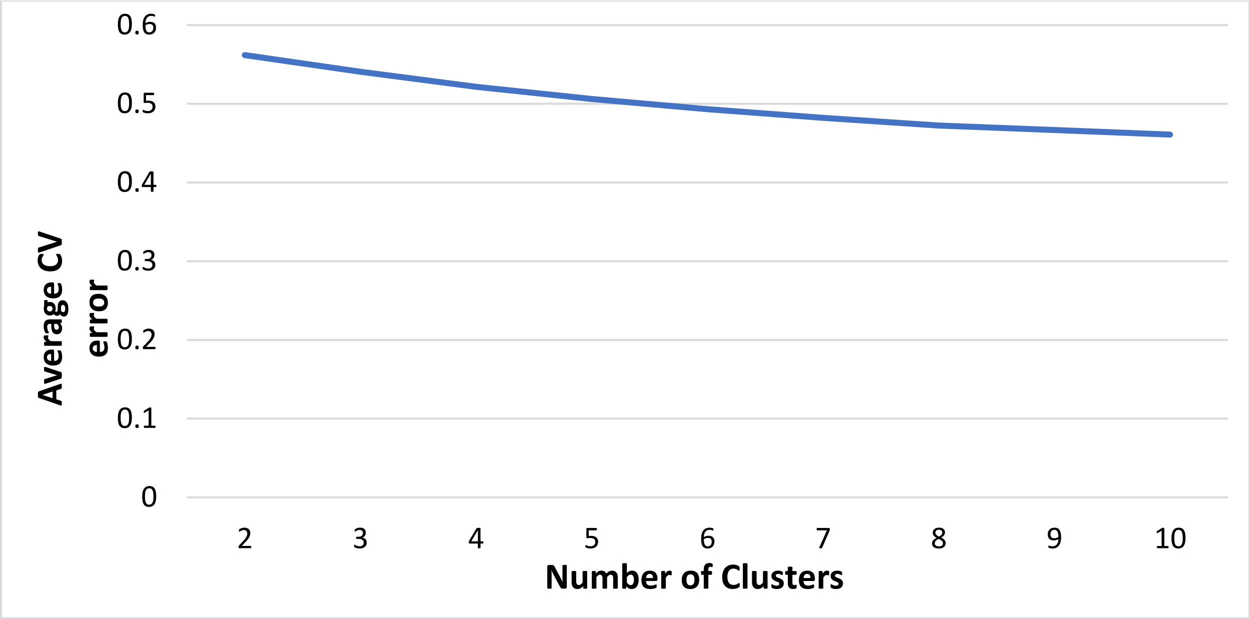

Supplement: Supplementary file 1 [file mmc1.pdf]
